# Web architecture, behavior, and predatory potential of *Larinia chloris* from rice fields (Araneae: Araneidae)

**DOI:** 10.1093/jisesa/iead030

**Published:** 2023-06-09

**Authors:** Sozaina Khan, Hafiz Muhammad Tahir, Abida Butt, Abbas Khan

**Affiliations:** Department of Zoology, Government College University, Lahore, Pakistan; Department of Zoology, Government College University, Lahore, Pakistan; Department of Zoology, University of the Punjab, Lahore, Pakistan; Department of Zoology, Government College University, Lahore, Pakistan

**Keywords:** rice, abundance, spider, insects, prey

## Abstract

Present study was carried out to investigate the variation in web architecture of *Larinia chloris* (Audouin 1826) in relation to seasonal differences and the biotic factors of the environment. In addition, relative abundance, behavior, and predatory potential of *L. chloris* were also recorded. For this purpose, 100 orb-webs of *L. chloris* were observed in rice fields (August–October, 2022) from 3 districts of Punjab (Lahore, Sheikhupura, and Kasur). Percent abundance of *L. chloris*was found to be highest in rice fields from Barki road, Lahore (39.53%). All the webs of *L. chloris* were vertical at height equal to the height of vegetation (115.2 ± 9.7 cm). Time required to complete the web was 45 ± 5 min. There was positive correlation between web architecture and vegetation height. Web capture area and average mesh height of *L. chloris* also showed positive correlation with carapace length. There was a significant difference in various web parameters (number of spirals, number of radii, capture area, average mesh height, upper radii, lower radii, left radii, and right radii) among different trapping months. A total of 1,326 insects were recorded from the 100 webs of *L. chloris*. The prey abundance was found to be highest in the fields from Barki Road, Lahore. The majority of the prey collected from webs of *L. chloris* belonged to order Diptera, Hemiptera, Coleoptera, and Lepidoptera. However, prey items recorded during different growth stages (from vegetative to ripening) varied significantly. This is the first ever report describing the ecology of *L. chloris* in rice fields from Punjab, Pakistan.

## Introduction

With approximately 50,531 known species in 117 families, spiders are the most abundant and ecologically important group of class Arachnida and order Araneae (World Spider Catalog 2022). They are fundamental part of terrestrial ecosystems with important practical applications as biocontrol agents and bio-indicators (Tahir et al. 2018, Ashfaq et al. 2019, Luqman et al. 2022). Orb weaving spiders are “sit and wait” predators as they employ unique foraging strategy by building web and are highly dependent on the web for their foraging activities (Vollrath and Samu 1987, Muhammad Nasir et al. 2017). The size and characteristic of orb webs vary depending upon the ecology of the species and are a direct reflection of the size and type of prey along with the capture efficiency (Jayakumar et al. 2017). They alter their web architecture in response to abiotic (weather, insecticides, and vegetation type) and biotic factors (predators, prey availability, intra- and inter-species competition, and animal disturbance) of the environment (Pasquet et al. 1994, Butt et al. 2017, Muhammad Nasir et al. 2017). Web characteristics also vary among individuals of same species (Pasquet et al. 2013). Different web features like web location, placement, size, capture area, web shape, the size and shape of decorations, silk properties, mesh height, and number of sticky threads influence the interception and retention rate of insect prey (Blamires 2010, Butt et al. 2017, Jayakumar et al. 2017). 

The size and characteristic of orb-webs vary depending upon the ecology of the species and are a direct reflection of the size and type of prey along with the capture efficiency (Jayakumar et al. 2017). For example, orb weavers of genus *Eriophora* build 2 dimensional webs in open areas and occupy the hub only during feeding. In contrast, spiders of the genus *Nephila* and *Tetragnatha* occupy hub all the time and form barrier webs or aggregate their webs in open habitat. Spiders of *Argiope* genus prefer to build smaller (hardly exceeding 0.5 m in diameter) webs among low, dense, and closed vegetation (Blamires et al. 2007). Generally, the orb-webs with narrow mesh are built by spiders for capturing small insects and the larger webs are likely to encounter more prey (Sandoval 1994, Schneider and Vollrath 1998, Heiling and Herberstein 2000). However, orb-weavers may relocate and alter their web architecture throughout their life time in response to abiotic and biotic factors of the environment (seasonal dynamic, insecticides, presence of spun lines, amount of available silk, vegetation type, nutrition, development stage, size and weight of spider, predators, prey availability, intra and interspecies competition, and animal disturbance) (Pasquet et al. 1994, Heiling and Herberstein 2000, Butt et al. 2017, Muhammad Nasir et al. 2017). These variations in web architecture enable the spiders to adapt and forage under varying environmental conditions (Blamires et al. 2007, Jayakumar et al. 2017, Phangurah 2019).

Orb weaving spiders are abundant in fields and regulate the pest populations in agro-ecosystems (Levi 1981, Nyffeler and Benz 1989, Aiken and Coyle 2000). Silk of some orb web spiders attracts large diversity of herbivorous insects. There may be up to 1,000 insects trapped in web at a given time and several are left in web to be consumed later (Tahir et al. 2009). The ecological and faunistic studies showed that orb-web spiders are common predators of the economically important pests and can significantly reduce pest densities below economic-injury level (Riechert and Lockely 1984, Nentwig 1986, Malik et al. 2018). They can be employed effectively to control pest population in major agricultural crops like wheat, rice, cotton, and maize (Manoley et al. 2003, Ghafoor and Mehmood 2011). Biological control using orb-web spider is one of the best strategies to control the pest populations and minimize the use of pesticides (Wise 1993, Ghaffor and Mehmood 2011, Anindita et al. 2017, Riaz et al. 2017, Somroo et al. 2022).

In the past few years, the use of pesticides has increased tremendously in agroecosystem especially in Pakistan. The vast use of insecticides have many disadvantages like long-term persistence, non-target specificity, bio magnification, and loss of biodiversity essential for ecological stability (Ghaffor and Mehmood 2011, Ghazanfar et al. 2016, Riaz et al. 2017). Keeping in view the negative impacts of pesticides, an integrated pest management program needs to be encouraged in order to conserve biodiversity and minimize the use of pesticides in crop fields. Despite the economic and ecological importance of orb-web spiders in agro-ecosystem, in Pakistan, only fragmentary work is available on the ecological role of spiders in pest control programs (Riaz et al. 2017, Sajid et al. 2021). There is a dire need to explore the biocontrol potential of orb-web spider by studying its web architecture and predatory activities (Tahir et al. 2011, 2015). The present study has been conducted to investigate the variation in web architecture of *Larinia chloris* in response to seasonal difference and biotic factors of the environment. Beside this, relative abundance, behavior, and predatory potential of female *L. chloris* (Audouin 1826) were also recorded in the rice fields from Punjab, Pakistan. *Larinia* has a worldwide distribution and includes 61 described species (World Spider Catalog 2022). This is the first report describing the variation in web architecture and predatory behavior of *L. chloris* in the rice fields from Punjab, Pakistan.

## Materials and Methods

### Field Surveys

**Fig. 1.**
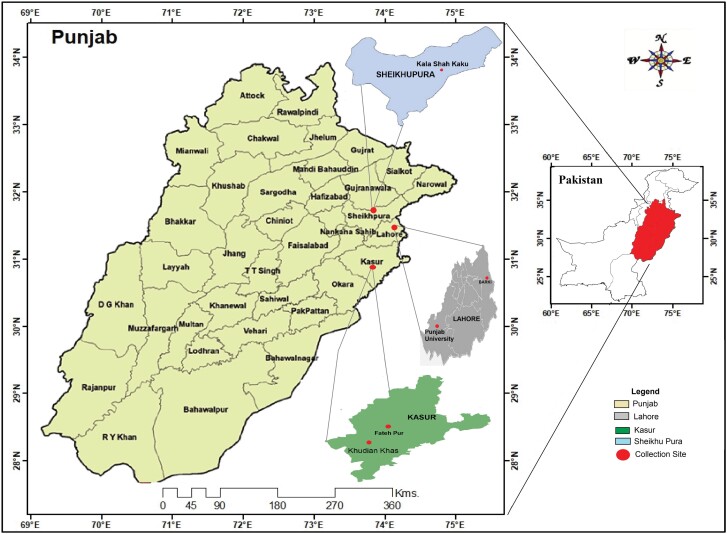
Map of Punjab, Pakistan showing different collection/study sites. Collection was carried out from the rice fields of 3 districts of Punjab, Pakistan (indicated by circle).

The study was carried out in the fields of rice (Basmati-86 and Basmati Super) during August–October, 2022 for the period of 3 months from 3 districts of Punjab (Lahore, Sheikhupura, and Kasur), Pakistan (Fig. 1). The study consisted a total of 30 surveys (6 surveys in each locality) throughout the growing season of rice from vegetative stage to ripening stage. Fields were surveyed during walk through an area of 400 m^2^ from 4 PM to 7 PM (PST) and all orb-webs present in area of 400 m^2^ were observed for spiders and their web architecture (all orb-webs in 400 m^2^ were examined to determine the abundance of *L. chloris* relative to other orb-web spiders present in rice fields from particular area) (Tahir et al. 2012, Butt et al. 2017). The plastic bags were used to capture and seize the spiders because they are very active and may be hazardous to humans (Khan and Zaman 2015). During sampling period, the information about temperature, humidity, and rainfall was recorded (Tahir et al. 2015, Halarnkar and Pai 2018, Mehmood et al. 2021). During the study period, average temperature varied from 26.4 °C to 30.9 °C and relative humidity from 73% to 58%.

### Relative Abundance of *Larinia chloris* (Audouin 1826)

Direct observation was made to determine the relative abundance of *L. chloris* in an area of 400 m^2^. The relative abundance of *L. chloris* at each locality was measured fortnightly. Relative abundance (%) at particular site was calculated by dividing the number of *L. chloris* specimens collected from particular locality by number of total collected specimens and result was multiplied with 100. Data of only females (juvenile and adults) was collected as males normally do not build webs (Wise 1993). Collected specimens were brought to the Applied Entomology and Toxicology Laboratory at Department of Zoology, Government College University Lahore, washed with 75% alcohol, and preserved in absolute alcohol until further processing (Sajid et al. 2021). Collected specimens were identified morphologically under dissecting microscope up to species level using identification keys and available catalog that is, Grasshoff (1970), Tikader and Malhotra (1980), Levy (1986), Tanikawa (1989), and other available literature.

### Morphometric Measurement of Web Architecture

**Fig. 2.**
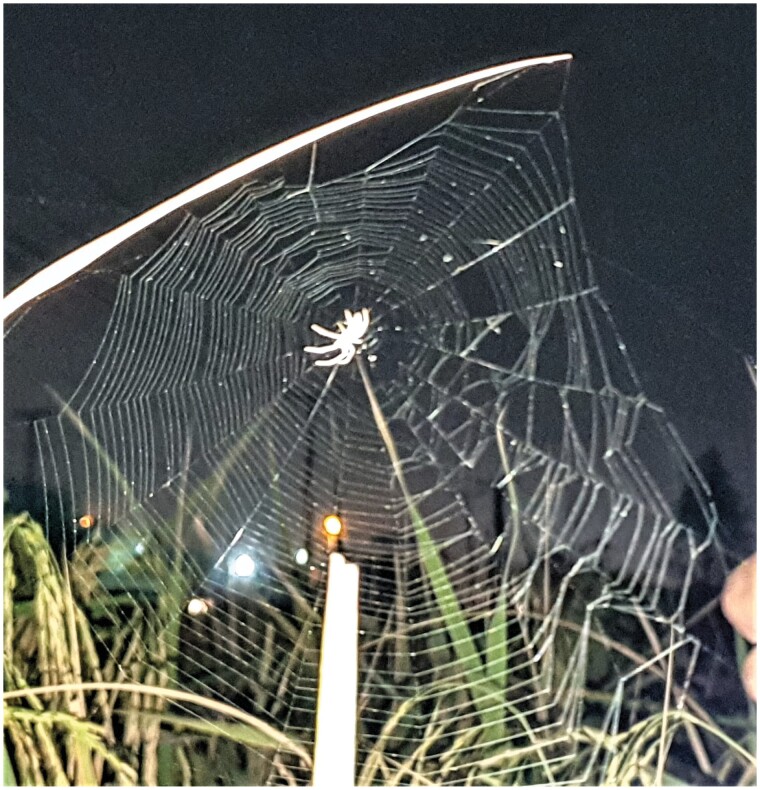
The orb web of *Larinia chloris* (Audouin 1826) collected from the rice field.


Web characteristics of the *L. chloris* were measured directly in the fields and recorded (McCravy and Hessler 2012, Muhammad Nasir et al. 2017). For measuring the web architecture, mist of water, and cornstarch was sprayed on each web to improve the resolution (Butt and Tahir 2010, Tahir et al. 2012) (Fig. 2). A total of 100 active webs of *L. chloris* were examined for its architecture. Following features were recorded in each web; height of the orb web, horizontal and vertical diameter, number of spirals, total number of radii, mesh size, hub size, free area, presence or absence of stabilimenta along with the size of stabilimenta and left, right, upper and lower halves radius from central axis (Vollrath et al. 1997, Blamires 2010, Tahir et al. 2010, Butt et al. 2017, Jayakumar et al. 2017). All the measurements were carried out by 10 m measuring tape (GW-F513W) (Jayakumar et al. 2017). Mesh size and capture area were calculated by the following formulae designed by Herberstein and Tso (2000) as these 2 parameters represent the change in web architecture and accordingly the prey capture ability (Vollrath and Samu 1987).

$$\begin{array}{l} \begin{array}{ll} & Capture\;area=\left[\frac{1}{2}\pi {r_{au}}^{2}-\frac{1}{2}\;\pi \;(Hr_{u}{)}^{2}\right]+\left[\frac{1}{2}\pi {r_{al}}^{2}-\frac{1}{2}\;\pi \;(Hr_{l}{)}^{2}\right]\\ & Average\;mesh\;height=\frac{1}{2}\left(\frac{r_{u}}{\left(S_{u}-1\right)}+\frac{Hr_{u}}{\left(S_{l}-1\right)}\right) \end{array} \end{array}$$

*r*_*au*_ = adjusted upper radii, *r*_*al*_ = adjusted lower radii, *Hr*_*u*_ = upper vertical hub radius, *Hr*_*l*_ = lower vertical hub radius, *S*_*u*_ = number of sticky spirals in the upper web half counted in vertical sector, *S*_*l*_ = number of sticky spirals in the lower web half counted in vertical sector.

Effect of seasonal variation and biotic factors on web building behavior of orb-web spiders was evaluated by comparing the web architecture under different conditions (months, plant height, and spider size) (McCravy and Hessler 2012, Muhammad Nasir et al. 2017).

### Morphometric Measurements of Orb-Web Spiders

For the evaluation of variation in web architecture in relation to size, the spiders were gently removed from the webs and morphometric measurements were taken using a Vernier caliper (Wijerathna et al. 2019). Size was recorded through the measurement of IV-leg length and carapace length/width using dissecting microscope and recorded (Lowe et al. 2014).

### Microhabitat (Vertical and Horizontal Distribution)

The vertical and horizontal distribution of the particular spider was determined by measuring the following; height of the web-the distance of web sheet from the ground and radius from the web hub to highest horizontal support. For each web, the height of plant to which web attach and height of the hub was recorded (Brown 1981, Muhammad Nasir et al. 2017).

### Prey Spectra

Direct observation were made to record the prey availability in the webs of the spider. Prey (alive, dead, partially eaten remains, or still in the possession of spiders) entangled in the web was identified morphologically up to order level in the field (Khuhro et al. 2020). The prey that could not be identified in the field were preserved in 70% ethanol and brought to the laboratory for identification (Butt and Tahir 2010, Tahir et al. 2012).

### Statistical Analyses

The normality of data was evaluated by Shapiro–Wilk test (Rodríguez-Rodríguez et al. 2015). Variations in web architecture in different collection months were investigated by general linear model (Vollrath et al. 1997). Spearman’s rank correlation was carried out to analyze the relationship among web attributes and spider size. Relation between web architecture and habitat features was also subjected to Spearman’s rank correlation. The probability level determining significant differences was *P* < 0.05 for all statistical tests. All these calculations were performed using statistical software, SPSS version 29.

## Results

**Fig. 3.**
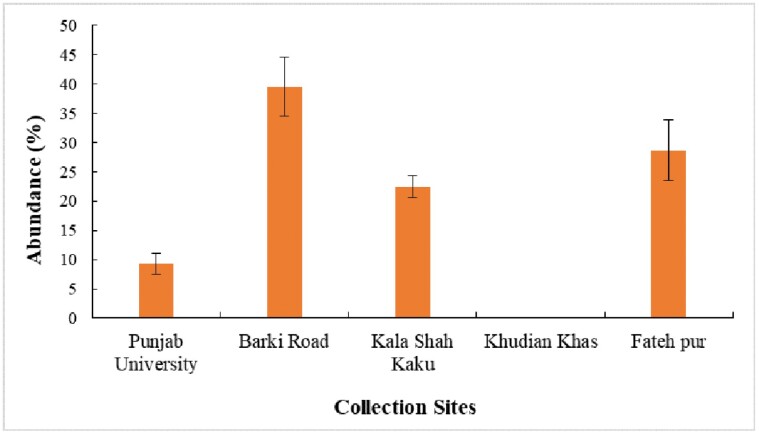
Percent relative abundance of *Larinia chloris* recorded from rice fields of 3 districts of Punjab, Pakistan. District Lahore: Punjab University (*n* = 12), Barki Road (*n* = 51). District Sheikhupura: Kala Shah Kaku (*n* = 29). District Kasur: Khudian Khas (*n* = 0), Fateh pur (*n* = 37). Error bars in the figure are representing Standard Error.

A total of 1,336 orb-webs were observed in different rice fields from 3 districts of Punjab, Pakistan. Out of the total 1,336 orb-webs studied, 129 webs belonged to *Larinia chloris* (Audouin 1826). Hundred (100) webs of *L. chloris* were included in this study (we excluded 29 webs which were either damaged, their webs architecture could not be measured, web was damaged, or web formation was in process). We selected only those 100 webs of *L. chlrois* which were perfect in all aspects. Data of both mature and immature female spiders was used as 75% orb-webs were constructed by adult and 25% by young females. Percent relative abundance (PRA) of *L. chloris* has been shown in Fig. 3. In Lahore district, the PRA of *L. chloris* was found to be highest (39.53%) in the rice fields of Barki road and lowest (9.3%) in the fields of Punjab University. No individual of *L. chloris* was recorded from the rice fields of Khudian Khas, Kasur. However, another area of Kasur (Fatehpur) was reported with 28.68% PRA of *L. chloris*. In the Kala Shah Kaku, District Sheikhupura, the abundance was found to be 22.48%.

*Larinia chloris* was less abundant in the month of August at each locality. It increased in the month of September (during reproductive stage) and highest peak was observed in late-October (ripening stage). Time required to complete the web was 45 ± 5 min. They build vertical webs between plants at the top of vegetation with close mesh spacing. *L. chloris* are nocturnal spiders; they build new webs daily at dusk and consume their webs in the morning. After building their webs, spiders were found at the hub of the web waiting for prey.

Different web characteristics recorded during the study period from August to October, 2022 are shown in Table 1. The web architecture was recorded to be altered in different months as analyzed by general linear model (Table 1). There were significant differences in various web parameters that is, number of spirals (*F*_2,42_ = 5.51; *P* = 0.02), number of radii (*F*_2,42_ = 9.224; *P* = 0.04), capture area (*F*_2,42_ = 5.713; *P* = 0.02), average mesh height (*F*_2,42_ = 5.51; *P* = 0.02), upper radii (*F*_2,42_ = 88.99; *P* = 0.004), lower radii (*F*_2,42_ = 4.256; *P* < 0.001), left radii (*F*_2,42_ = 6.511; *P* = 0.012), and right radii (*F*_2,42_ = 5.704; *P* < 0.018) between 3 trapping months (August, September, and October). Spiders responded to seasonal dynamics by increasing overall capture area. However, the free area was not be found to be altered significantly (*F*_2,42_ = 2.351; *P* = 0.138).

**Table 1.** Variation in web architecture (cm) during study period analyzed by general linear model

Month	August	September	October	*F*	*P*
Number of spirals	24.8 ± 1.98	37.8 ± 4.5	35.4 ± 1.2	5.51	0.02
Number of radii	15.6 ± 0.81	19.6 ± 1.0	17.8 ± 0.58	5.574	0.019
Capture area	20.5 ± 3.6	127.44 ± 26.4	231.23 ± 53.6	9.244	0.04
Average mesh height	0.087 ± 0.009	0.144 ± 0.018	0.219 ± 0.03	5.713	0.018
Lower radii	2.4 ± 0.24	7.0 ± 0.89	10.4 ± 1.11	23.084	<0.001
Upper radii	2.7 ± 0.30	4.7 ± 0.37	5.7 ± 0.75	8.899	0.004
Left radii	3.8 ± 0.37	7.9 ± 0.871	8.4 ± 1.4	6.511	0.012
Right radii	3.5 ± 0.35	8.0 ± 1.1	8.2 ± 1.4	5.704	0.018
Free area	1.74 ± 0.166	2.6 ± 1.1	0.65 ± 0.100	2.351	0.138

All the webs of *L. chloris* were vertical at height equal to the height of vegetation (115.2 ± 9.7). The data about the web architecture and web site features has been shown in Table 2. The web is divided into 2 main regions; horizontal and vertical diameter. Horizontal length of the capture area varied from 5 to 24 cm, vertical length varied from 3.5 to 22.5 cm, and web circumference ranged from 15.7 to 75.36 cm^2^. Similarly the plant height and web height were in the range of 54–160 cm and distance from hub to horizontal support varied between 4 and 50 cm. Results of Spearman’s rank correlation showed the positive correlation of plant height with (i) horizontal length of the capture area (*r* = 0.747; *P* = 0.001), (ii) vertical length of the capture area (*r* = 0.710; *P* = 0.003), (iii) web circumference (*r* = 0.747; *P* = 0.001, (iv) web height (*r* = 1.000; *P* < 0.001), (v) hub height (*r* = 0.998; *P* < 0.001) and distance from hub to horizontal support (*r* = 0.698; *P* = 0.004), and average mesh height (*r* = 0.568; *P* = 0.027) (Table 3).

**Table 2.** Web and web site features of *L. chloris* in the various rice fields

Attributes (cm)	Average ± SE	Minimum	Maximum
Web characteristics			
Horizontal length of capture area	13.26 ± 1.5	5	24
Vertical length of capture area	10.9 ± 1.39	3.5	22.5
Web circumference	41.65 ± 4.8	15.7	75.36
Average mesh height	0.155 ± 0.20	0.06	0.36
Web site features			
Plant height	115.2 ± 9.7	54	160
Web height from ground	115.2 ± 9.7	54	160
Hub height from ground	110.88 ± 9.4	52	154
Distance from hub-horizontal support	20.8 ± 3.5	4	50

**Table 3.** Correlation matrix showing the relationship between web architecture and vegetation height

Web and web site features		Horizontal length of capture area	Vertical length of capture area	Web circumference	Web height from ground	Hub height from ground	Distance from hub-horizontal support	Average mesh height
Plant height	*r* value	0.747**	0.710**	0.747**	1.000**	0.998**	0.698**	0.568*
	*P* value	0.001	0.003	0.001	<0.001	<0.001	0.004	0.027

*Correlation is significant at the 0.05 level (2-tailed).

**Correlation is significant at the 0.01 level (2-tailed).

The variations in web architecture in relation to different body measures (Table 4) of the spider were analyzed by Spearman’s rank correlation (Table 5). Web architecture was related differently to different body measures. There was positive correlation between carapace length and (i) capture area (*r* = 0.808; *P* < 0.001), (ii) average mesh height (*r* = 0.855; *P* < 0.001) (iii) vertical diameter (*r* = 0.814; *P* < 0.001), and horizontal diameter (*r* = 0.823; *P* < 0.001). Carapace width was not related to capture area (*r* = 0.121; *P* = 0.661), vertical diameter (*r* = 0.122; *P* = 0.666), horizontal diameter (*r* = 0.505; *P* = 0.055), and average mesh height (*r* = 0.259; *P* = 0.351). The increase in leg IV of *L. chloris* female did not alter the web capture area (*r* = 0.346; *P* = 0.207) and average mesh height (*r* = 0.414; *P* = 0.125) but showed positive correlation with vertical diameter (*r* = 0.607; *P* = 0.016) and horizontal diameter (*r* = 0.719; *P* = 0.003).

**Table 4.** Various Body measures (means ± SE) of *L. chloris* collected from rice fields

Body measures	Average ± SE	Minimum	Maximum
Carapace length	9 ± 0.51	4.5	11.5
Carapace width	2.48 ± 0.099	1.5	3
4th leg measurement	11.4 0.619	5.5	13.25

**Table 5.** Correlation coefficient values for body measurements and web parameters of adult female of *L. chloris* spider (*P < 0.05; **P < 0.01)

	Average mesh height	Capture area	Vertical diameter	Horizontal diameter
Carapace length	0.855**	0.808**	0.814**	0.823**
	<0.001	<0.001	<0.001	<0.001
Carapace width	0.259	0.121	0.122	0.505
	0.351	0.661	0.666	0.055
IV leg length	0.478	0.611	0.607*	0.719*
	0.071	0.016	0.016	0.003

*Correlation is significant at the 0.05 level (2-tailed).

**Correlation is significant at the 0.01 level (2-tailed).

During study period, a total of 1,326 insects were recorded from 100 webs of *L. chloris* (Table 6). The abundance of insects prey recorded from the webs of *L. chloris* at various collection points varied greatly. Highest number of insects were recorded from the fields of Barki Road, Lahore (*n* = 482). However, the lowest number was observed from the webs at Punjab University (*n* = 167). In addition, insect density also varied during different growth stages with lowest number in vegetative stage and reaching to maximum in reproductive stage. Insects belonging to 4 different orders constituted the main prey spectra (Table 7). However, dominant prey orders varied during different stages of crop. During vegetative stage, the prey belonging to order Diptera dominated the prey spectra followed by Hemiptera, Coleoptera, and Lepidoptera. Individuals of order Hemiptera were recorded from majority of webs in reproductive stage, followed by Diptera, Lepidoptera, and Coleoptera. In the reproductive stage, density of insect prey increased rapidly followed by decline during ripening stage. In the 3rd stage of rice growth (ripening stage), the order Diptera is over-represented and Lepidoptera is under-represented as main prey in the webs of *L. chloris.*

**Table 6.** Total number (N) of *L. chloris* and prey (from webs) recorded at different stages of the rice from 3 districts of Punjab, Pakistan

Growth stage	Punjab University		Barki Road		Kala Shah Kaku		Khudian Khas		Fateh pur		Total	
	S	P	S	P	S	P	S	P	S	P	S	P
Vegetative stage	1	27	7	49	6	23	0	0	3	34	17	133
Reproductive stage	4	87	21	277	12	187	0	0	13	211	50	762
Ripening stage	7	53	23	156	11	102	0	0	21	120	62	431
Total	12	167	51	482	29	312	0	0	37	365	129	1,326

S, spider, P, insect prey.

**Table 7.** Number of insect prey order captured by spider webs at different growth stages of rice crop

Prey orders	Number of prey in webs (mean ± SE)		
	VEG*	REP**	RIP***
Diptera	18.33 ± 1.6	67.33 ± 3.71	58.66 ± 5.33
Hemiptera	9.33 ± 0.88	89 ± 3.0	26.66 ± 2.66
Coleoptera	9.33 ± 0.88	38.66 ± 1.20	31.33 ± 4.66
Lepidoptera	6 ± 1.7	40.33 ± 1.20	10.66 ± 2.66
Unidentified	1.33 ± 0.3	18.66 ± 1.20	16.33 ± 2.0

*Vegetative stage. **Reproductive stage. ***Ripening stage.

## Discussion

The abundance of *L. chloris* was determined from surveys of rice fields throughout the growth period. The growth stage of rice plant affected the abundance of *L. chloris*. The abundance was lowest in the vegetative stage (August) and highest in the ripening stage (late October); however, the differences among the 3 seasons were not statistically significant. The results are in accordance with the previous studies that reported the lowest abundance of orb web spiders in vegetative stage and higher in other stages of rice growth (Wilson et al. 2014, Tsutsui et al. 2016, Saksongmuang et al. 2020).

The difference in abundance of *L. chloris* was related to the density of insects prey in different growth stages as highest numbers of insects were recovered from webs during reproductive stages. The higher abundance in the reproductive stage could be related with the high abundance of insect prey due to wet conditions and appearance of flowers in rice crops as suggested by previous studies (Zhi-yu et al. 2011, Takada 2014, Saksongmuang et al. 2020). The flooded paddy rice provides habitat for variety of aquatic insects like midges, flies, pest (armyworms, planthoppers, whorl maggot), and various types of bugs. The flowers of rice in the reproductive stage are also the source of wide range of insects (Saksongmuang et al. 2020). In addition, the highest abundance of insect prey in the fields of Barki Road could also be the reason of highest spider abundance at this site. Beside this, abundance of *L. chloris* was observed to be varied in different fields with the availability and length of water bodies as abundance of spider was higher either in fields at the bank of water bodies or in fields with long water canal.

During study period, the overall capture area and average mesh height increased significantly from August to October, 2022. It could be possible that the variation in temperature and humidity effected the physiology of *L. chloris* and ultimately web-building capability. Previous studies reported the variation in web architecture in response to altered temperature and humidity but their interaction was not understood in the field. Vollrath et al. (1997) reported that the decrease in temperature from 24 °C to 12 °C increased spiral spacing of *Araneus diadematus* spider in an electronic-controlled climate cabinet and web area was not altered overall. Boutry and Blackledge (2013) examined the influence of humidity on prey capture performance. It was reported that webs at high humidity (>70% RH) intercepted prey better without breaking than those at low humidity (30–35% RH). Major ampullate silk incorporated in orb web undergoes changes in mechanical properties when exposed to ecologically high temperatures and low humidity, with temperature increasing its strength and stiffness (Blamires and Sellers 2019). Further studies are required to evaluate the variation in web architecture of *L. chloris* in response to a range of temperature and humidity.

The location of a spider web within its habitat often conveys important information about the behavior and ecology of the spiders (Jayakumar 2017). All the observed spiders of *L. chloris* were nocturnal and build vertical webs at top of the vegetation near the water body. Most of the spiders observed (75%) build webs at height above 100 cm, thus possibly reducing the interspecies competition. There was positive correlation with plant height and web height. In addition, web size (capture area, web circumference, average mesh height, vertical, and horizontal length, height of hub) also increased with increase in vegetation height, thus more open web with large capture area might be an adaptation to capture more prey thus increasing feeding efficiency. High and large web capture more insects especially large flying insects as described by previous studies (Bishop and Connolly 1992, Eberhard 1992, Tahir and Butt 2010).

In present study, web architecture varied with the various body measures. Capture area and average mesh height increased with increase in carapace length but did not show any relationship with carapace width. Heiling and Herberstein (1988) reported no relationship between mesh size and any body size measurements in adults of *Nuctenea sclopetaria*. However, earlier studies reported the positive correlation of carapace width with capture area (Olive 1980, Heiling and Herberstein 1988, Tahir and Butt 2010, Butt and Alam 2017). The leg IV length did not show any relation with capture area and average mesh height as observed by Tahir et al. (2009). However, several studies reported the positive relation between leg IV length and web architecture (Eberhard 1988, Butt and Alam 2017). In the present study, the carapace length was most appropriate variable to indicate the effect of body size on web architecture.

The highest prey abundance at the fields from Barki road favored the high abundance of *L. chloris* as suggested by previous studies that the high prey abundance increased the numerical response of spider (Wise 1979, Saksongmuang et al. 2020). In addition, in the early cropping season, insects of order Diptera dominated the prey spectra. This is in consistence with the previous studies where nonpest dipterous insects were the most numerous prey consumed in the early growing season and strengthen the top down effect for thriving the pest during reproductive stage (Ishijima et al. 2006, Motobayashi et al. 2006, Tahir and Butt 2010, Tsutsui et al. 2016). In the reproductive stage, conditions become suitable for the herbivorous pests to thrive and insects belonged to Hemiptera and Lepidoptera were found in the webs of *L. chloris*. Insects flourished to peak during the reproductive stage due to availability of habitat and food in accordance to previous studies by Kiritani et al. (1972), Ishijima et al. (2004), and Saksongmuang et al. (2020). However, this is in contrast to the findings by Tahir and Butt (2010), who reported the diptera as representative of prey spectra during ripening phase. In the ripening stage, number of insect prey recorded in the webs declined greatly since the rice plant become too hard and dense for feeding by sucking insects as suggested by Saksongmuang et al. (2020). However, Hemiptera and Diptera represented the major prey spectra throughout the growth period of rice.

In this study, *L. chloris* was found to be more abundant in the rice fields of Barki road, Lahore. Web architecture varied significantly during study period (from August to October 2022). In addition, it was correlated positively with carapace length and plant height. The availability of insect prey in the fields affected the numerical response of *L. chloris*. Prey spectra were characterized by 4 major insect groups (Diptera, Hemiptera, Coleoptera, and Lepidoptera), most of which represented by insect pests. *L. chloris* was found to be feeding on these insect pests. The current research would provide back ground knowledge for developing strategy for sustainable pest control with low cost, environment friendly, and suitable for implementation in developing countries like Pakistan.
